# Understanding cellular internalization pathways of silicon nanowires

**DOI:** 10.1186/s12951-017-0250-0

**Published:** 2017-03-01

**Authors:** Kelly McNear, Yimin Huang, Chen Yang

**Affiliations:** 1 Department of Chemistry, Purdue University, 560 Oval Drive, West Lafayette, IN 47907 USA; 2 Department of Physics and Astronomy, Purdue University, West Lafayette, IN 47907 USA

**Keywords:** Cellular interaction, Silicon nanowires, Membrane wrapping

## Abstract

**Background:**

Understanding how cells interact with nanomaterials is important for rational design of nanomaterials for nanomedicine and transforming them for clinical applications. Particularly, the mechanism for one-dimensional (1D) nanomaterials with high aspect ratios still remains unclear.

**Results:**

In this work, we present amine-functionalized silicon nanowires (SiNW-NH_2_) entering CHO-β cells via a physical membrane wrapping mechanism. By utilizing optical microscopy, transmission electron microscopy, and confocal fluorescence microscopy, we successfully visualized the key steps of internalization of SiNW-NH_2_ into cells.

**Conclusion:**

Our results provide insight into the interaction between 1D nanomaterials and confirm that these materials can be used for understanding membrane mechanics through physical stress exerted on the membrane.

**Electronic supplementary material:**

The online version of this article (doi:10.1186/s12951-017-0250-0) contains supplementary material, which is available to authorized users.

## Background

As nanotechnology advances as an innovative option in clinical settings, researchers continue to explore a wide array of nanomaterials for applications as imaging and anti-cancer agents, for drug delivery purposes, and for therapeutics. While this progress has been exciting for the future of medicine, these materials have not overcome the barrier of translating from benchtop to clinic. In order for nanomaterials to advance as viable options for biological applications, further understanding of the basic interactions between mammalian cells and nanomaterials must be achieved.

In the past few decades, to understand how the cellular membrane can respond to the entry of external nanomaterials research has been mainly focused on finding the endocytosis pathways of zero dimensional (0D) nanomaterials [1–5]. Limited efforts have also been made to understand the uptake of various 1D nanomaterials into cells. For example, gold nanorods and magnetic nanowires have been heavily studied for imaging and tracking purposes [3, 6–8], but their specific uptake pathways were not well-studied. Single-walled carbon nanotubes (SWCNTs) have also been of interest due to their high aspect ratio and uniqueness as a material. Yaron and coworkers showed that the uptake of SWCNTs was energy-dependent, suggesting that the pathway is endocytosis and not membrane penetration, but the surface and size is so dissimilar to that of other longer 1D materials that these uptake pathways cannot be translated [9]. Additionally, Kostarelos and co-authors investigated previously investigated functionalized carbon nanotubes (*f*-CNTs) with a variety of functional groups, including an ammonium functionalization. The authors observed that the ammonium functionalized wires enter the cells at both 37 and 4 °C, ruling out a receptor-mediated pathway [10]. Notably, the dimensions of the *f*-CNTs that were studied were on the scale of 1 nm in diameter and 1000 nm in length, making them unique from other 1D nanomaterials.

Theoretical studies on both 0D and 1D nanostructures suggest that understanding the membrane mechanics during endocytosis is a critical aspect to explain internalization pathways of these nanostructures. Huang and co-authors designed a nanorod model using coarse-grained molecular dynamics to demonstrate that for endocytosis such model system needs to initially bind in an upright docking position, on the membrane plane, and then be wrapped by the membrane in order to proceed through a laying-down-then-standing-up sequence to enter cells [11]. Based on this work, it is reasonable to believe that membrane wrapping occurs as one of the first steps in endocytosis. Shi and co-authors used multi-walled carbon nanotubes to experimentally and theoretically illustrate that the cell entry of 1D nanomaterials can occur by tip recognition and rotation, but the authors do not delve into the details of internalization [12]. Yi and co-workers theoretically proved that cell membrane internalizes 1D nanomaterials following a near-perpendicular entry mode at small membrane tension but switches to a near-parallel interaction mode at large membrane tension [13]. These theoretical models illustrate the necessity to experimentally understand the membrane interactions of high aspect ratio 1D nanomaterials.

Due to the advantages of anisotropy and higher surface area to volume ratios than 0D nanomaterials, 1D nanomaterials can produce a stronger interaction with cells during the entering process. These features indicate that 1D nanomaterials can be considered as a better system to explore the possible membrane wrapping mechanism in the uptake pathways of nanomaterials [14, 15]. To this end, in this work we use multiple microscopy methods to visualize key steps during the cellular internalization process of silicon nanowires. Since silicon nanowires are fabricated with a complete control of dimensions [16, 17], have flexible chemistry for surface modification [18], have unique optical properties for in vitro and in vivo imaging [19–21], they are excellent candidates for the studies of cellular uptake pathways. Silicon nanowires modified with amine groups are the focus of the study, as compared to as-grown SiNWs with hydroxyl groups and SiNWs with specific targeting groups conjugated, SiNW-NH_2_ are able to be internalized in cells without targeting receptor mediated processes [22]. We demonstrate the uptake pathway of 5 µm SiNW-NH_2_ to be a physical membrane wrapping mechanism using CHO-β and HeLa cell lines. Studies at two different incubation temperatures, 37 and 4 °C, were carried out in order to evaluate temperature dependence of the membrane mechanics as well as to elucidate that the process is physically driven rather than receptor-mediated. We chose 4 °C because it is well understood that many endocytic pathways are temperature dependent and that these pathways are limited to high temperature due to the large activation barrier, so uptake at a lower temperature would indicate that the mechanism is physically driven [23].

## Methods

### Synthesis and functionalization of SiNWs

Silicon nanowires were synthesized by chemical vapor deposition (CVD) with 40 nm gold nanoparticles as growth catalysts and silane as a precursor. SiNWs in length of 5 µm were chosen in this works because of the appropriate aspect ratio, low cytotoxicity and considerable high endocytosis rate to study the cellular internalization [22]. The CVD growth was carried out in with a growth pressure of 100 Torr, silane flow rate of 5 sccm, and growth time of 5 min [22]. A representative TEM micrograph (Additional file 1: Figure S1) shows that the dimensions of the wires are consistent with the growth conditions.

The as-grown SiNWs were first treated with thermal oxidation in atmosphere at 900 °C for 2 min in order to clean and oxidize the surface for further modification. The thermally oxidized SiNWs were then submerged in a solution of 1% (3-aminopropyl) trimethoxysilane (APTMS) (Sigma-Aldrich, St. Louis, MO, USA) in pure ethanol overnight at room temperature. The substrates were then rinsed and submerged in pure ethanol at 80 °C in order to stabilize the functional groups. After 2 h, the substrates were removed and dried under a stream of nitrogen. SiNWs-NH_2_ were then removed from the substrate into pure ethanol via sonication for several seconds. The SiNW-NH_2_ suspensions were centrifuged at 14,000 rpm three times to wash away impurities. The wires were then resuspended in a complete Roswell Park Memorial Institute (RPMI) 1640 cell culture medium with 10% FBS, 1% l-glutamine, 1% penicillin/streptococcus for future use.

In order to confirm that the amine modification was successful, unmodified SiNWs and SiNWs-NH_2_ were sonicated in ultrapure water and placed in disposable folded capillary cells for zeta potential measurements. The concentration of SiNWs and SiNW-NH_2_ in each capillary cell was estimated to be 10 µg/mL. Measurements were carried out on a Malvern Zetasizer Nano Series.

To further confirm and quantify amine functionalization on the modified silicon nanowires, the authors performed Fourier transform infrared spectroscopy (FTIR) (Thermo Nicolet Nexus FTIR) as well as X-ray photoelectron spectroscopy (XPS) analysis. As grown nanowires on growth substrates were modified with amine and used as the modified samples for measurements here. Comparison between the FTIR spectrum for the unmodified silicon nanowires (blue curve in Additional file 1: Figure S2) and the FTIR for the amine-modified wires (red curve in Additional file 1: Figure S2) shows that a peak near 1600 cm^−1^ in the spectrum for the amine-modified wires is visible and corresponds to the NH_2_ bending mode, confirming the presence of amine groups. The XPS spectrum measured from the modified SiNW-NH_2_ sample (Additional file 1: Figure S3) shows atomic percentages of N and Si of 1.2 and 20.3%, respectively. Based on this result, the amine coverage was estimated to be 0.61 mol/nm^2^, which is close to the coverage previously modeled based on the total covalently bonded APTES coverage on silica [24].

### Cell culture

We cultured immortalized Chinese hamster ovary cells transfected with folate receptor beta (CHO-β) for our cellular interaction studies. Our previous study investigated the uptake of SiNW-NH_2_ using both CHO and CHO-β cells. The findings showed that SiNW-NH_2_ were successfully internalized by both cell types. Therefore, CHO-β was chosen as the cell line of choice due to their success in internalizing SiNW-NH_2_ [22]. Typically, CHO-β cells were cultured in the complete RPMI 1640 mentioned above at 37 °C in a humidified atmosphere with 5% CO_2_. Cell viability was maintained and confirmed by cell morphology under optical microscopy during the testing periods discussed in the work [25, 26]. For optical studies, the CHO-β cells were cultured on sterile glass cover slides in 35 × 10 mm tissue culture dishes with one million cells per milliliter. Once they came to confluency, the cells were ready for microscopy studies.

### Optical microscopy

To prepare samples for the optical images, the medium was removed from the culture dish and the dish was rinsed with 1 mL of phosphate buffered saline (PBS). 1 mL of the prepared nanowire solution was added to the dish and the cells were incubated with the wires for various time points.

Before imaging, the SiNW-NH_2_ solution was removed and the dishes were washed with 1 mL PBS. To achieve better focus of live cells, we prepare samples as follows. A microscope slide was prepared by adhering double sided tape along the long edges of the slide. The glass cover slide taken from the cell dish was then placed on top of the prepared microscope slide. One side of the cover slide was sealed with nail varnish. Fresh medium was added between the cover glass and the slide followed by sealing the other side. Bright field, fluorescent, and dark field images were collected using an Olympus BX-51 optical microscope.

### Transmission electron microscopy

Once the cells were incubated with the wires for a given amount of time, the nanowire solution was removed via pipette and rinsed with 1 mL of PBS. The PBS was replaced with 2 mL of 2.5% glutaraldehyde and 0.1 M sodium cacodylate buffer solution (fix). This solution was allowed to sit for several minutes before being poured off. Another 2 mL of the fix was added and the cells were scraped from the bottom of the dish and transferred to a small conical centrifuge tube. The cells were spun down, the old fix was removed and 1.5 mL of fresh fix was added.

The remainder of the processing was done at the Purdue Life Sciences Microscopy Facility. Cells were embedded in 2% agarose, and post-fixed in buffered 1% osmium tetroxide containing 0.8% potassium ferricyanide. Cells were then *en bloc* stained in 4% uranyl acetate, dehydrated with a graded series of ethanol, and embedded in LX-112 resin. Sections with a 90 nm thickness for the 37 °C and 180 nm for the 4 °C samples were cut using a Reichert-Jung Ultracut E ultramicrotome and stained with 2% uranyl acetate and lead citrate. Images were acquired on a FEI Tecnai G220 electron microscope equipped with a LaB6 source and operated at 100 kV.

### Confocal microscopy

After incubation, the SiNW-NH_2_ solution was removed and the dishes were washed twice with 1 mL PBS. PBS was removed and 1 mL of 5 µM 1,1′-dioctadecyl-3,3,3′,3′-tetramethylindocarbocyanine perchlorate (DiI) was added and allowed to incubate at 37 °C for 20 min. The DiI was then removed and the dish was rinsed twice with 1 mL of PBS. PBS was replaced with 2 mL of fresh culture medium and the cells were incubated at 37 °C for another 10 min to rinse off excess DiI. The old medium was removed before cells were imaged.

The glass cover slide was placed on a microscope slide using the aforementioned preparation. Images were taken using an Olympus FluoView 300 Confocal Laser Scanning Microscope with a 543 nm excitation. For each area, approximately 100 images were taken at 10 µm steps through the Z-axis and the images obtained towards the center of the top and bottom of the cells are presented here.

## Results and discussion

### Zeta potential measurement confirms successful surface functionalization

The as-grown SiNWs were surface modified with amine groups to introduce a positive surface charge. The zeta potential measurements were carried out in triplicate to confirm the success of functionalization. The zeta potential of the unmodified SiNWs was measured to be −29.7 ± 7.85 mV, due to the oxide layer on the SiNW surface. The zeta potential for the SiNW-NH_2_ was measured to be +28.1 ± 5.11 mV, consistent with that the amine groups are protonated under neutral conditions. Such change in zeta potential after the modification confirms a successful functionalization. Since the cell membrane has slightly negative charge [27], the positive charge of the SiNW-NH_2_ will ensure charge–charge interaction between the wires and cell membrane.

### The interaction between SiNW-NH_2_ and cells is insensitive to temperature

As we previously reported [22], no obvious binding or internalization was observed in the CHO-β cells treated with the same concentration of unmodified SiNWs even after 11 h incubation (see Additional file 1: Figure S4). We attributed the observation of no interaction to the lack of charge–charge interaction between the unmodified SiNWs and the cell membrane [22]. This is the also the reason why we chose to focus on amine-functionalized SiNWs in this paper.

**Fig. 1:**
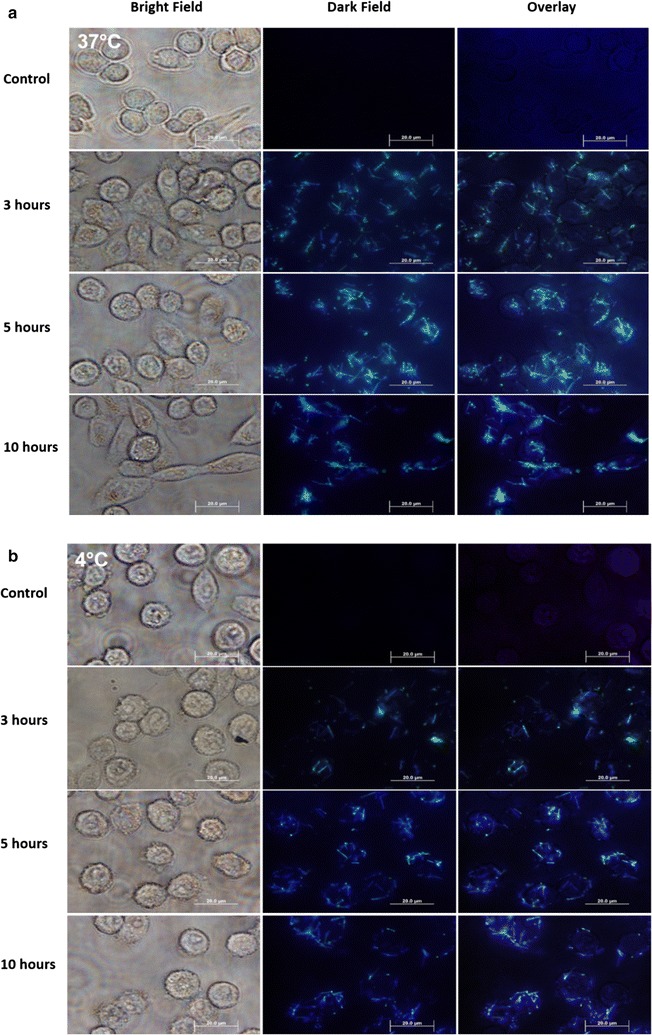
Optical microscopy images of SiNW-NH_2_ after **a** 37 °C incubation and **b** 4 °C incubation (*bottom*) with CHO-β cells. The images of control group, after 3 h of incubation group, after 5 h of incubation group, and after 10 h of incubation group are displayed, respectively. *Scale bars* 20 µm

Bright field and dark field transmission optical imaging was utilized not only to confirm the interaction of the SiNW-NH_2_, but also to investigate if temperature played a role in membrane interaction. The bright field images in Fig. 1 were used to visualize the cells, while the dark field images were taken to confirm the presence of the SiNW-NH_2_. SiNW-NH_2_ were co-cultured with cells at both 37 and 4 °C for 3, 5 and 10 h, respectively. PBS was used to wash the cells prior to imaging, so any wires in the medium or weakly interacting wires were removed. Notably, due to the limitation of the optical transmission imaging, bound wires and internalized wires cannot be differentiated, therefore both scenarios were considered as the wires interacting with the cells. To directly observe the internalized wires, we also used the TEM and confocal microscopy imaging and results are presented later in this manuscript. The control groups were cells incubated at 37 and 4 °C without the presence of wires for 3 h. As shown in Fig. 1a, after incubation for 3 h at 37 °C, SiNW-NH_2_ were observed binding to the membrane of the cells. As the incubation time increased, more wires could be seen interacting with the cells. Figure 1b depicts the wire interactions at 4 °C and we noticed similar trends of interaction, albeit with less wires observed than the 37 °C group. Binding was also observed after the 3-h incubation period in both the 37 and 4 °C optical images, a slight increase in the number of wires interacting with the cells can be seen in the 5 h panels of both the 37 and 4 °C optical images, and many wires were still visible in the image after 10 h.

**Fig. 2:**
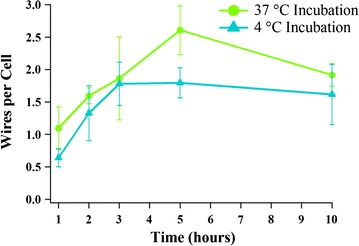
Statistical representation of the numbers of wires interacting per cell as a function of incubation time at 37 °C (*green*) and 4 °C (*blue*). *Error bars* are standard deviations measured from 5 sample areas for each time point

Figure 2 plotted the quantitative analysis of wires interacting with cells as a function of incubation time at 37 and 4 °C. Wires were counted from the overlay of the bright and dark field images. Five areas measuring 70 µm × 50 µm were examined for each sample. Numbers of wires interacting per cell measured and standard deviation of the data presented as error bars are plotted in Fig. 2. Figure 2 showed similar trends for both temperatures as functions of the incubation period with more wires interacting with cells at 37 °C. The average rate of interaction over the first 5 h of the incubation period was estimated to be 0.5 wires per cell per hour at 37 °C (green curve), while 0.4 wires per cell per hour at 4 °C, likely indicating a slightly slower internalization for 4 °C. Such small difference can be attributed to the fact that the cells incubated at 4 °C were not as active as they were at 37 °C. It is well-known that receptor mediated endocytosis processes do not happen at low temperatures [28–30]. A small decrease in the number of wires interacting with cells after 5 h at 37 °C was observed. We attributed the decrease after 5 h in the number of wires interacting with cells to exocytosis, as it is consistent with the reported values between 6 and 8 h at 37 °C for the typical time scale for exocytosis of nanoparticles and nanorods [29]. Since it is a competing process between endocytosis and exocytosis once the SiNWs enter the cells, we attributed the decrease in the number of wires interacting with cells after 5 h at 37 °C to exocytosis. The interaction between SiNW-NH_2_ and cells was found to be insensitive to temperature in our studies, indicating that the charge–charge attraction induced interaction between SiNW-NH_2_ and cells is not through the receptor mediated pathway but a physically driven process.

### Cross-section TEM images indicate membrane wrapping

**Fig. 3:**
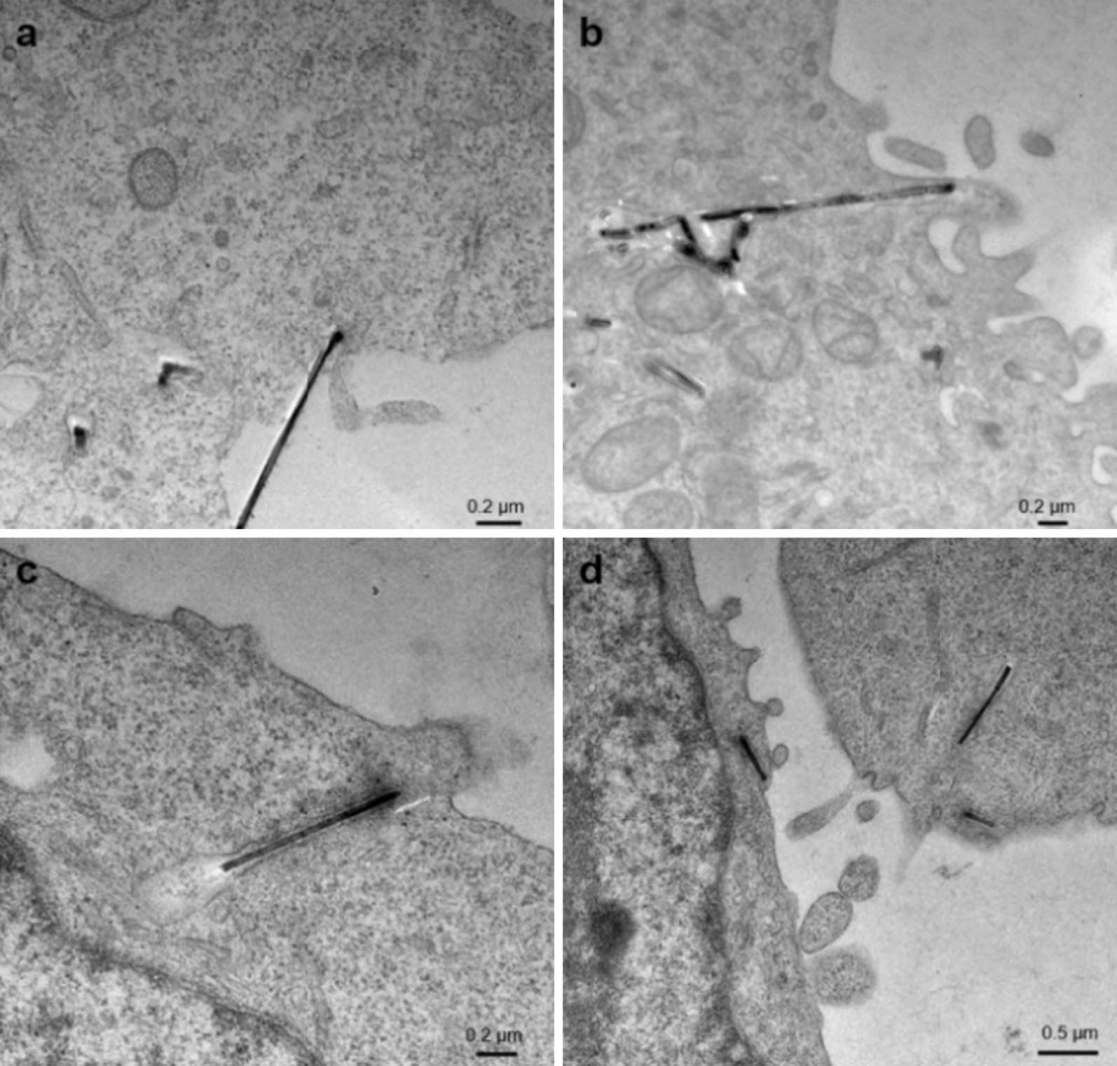
Cross section TEM micrographs represent CHO-β cells incubated with 5 µm wires for **a** 2, **b** 3, **c** 5, and **d** 10 h at 37 °C. *Scale bars* are 0.2 µm in **a**–**c** and 0.5 µm in **d**, respectively

We utilized cross-sectional TEM studies to visualize the relative position of bound and internalized nanowires to cell membrane. Specifically, cross-sectional TEM images can confirm that the SiNW-NH_2_ are internalized by the cells and can be used to examine how the membrane of the cells interacts with the wires, offering insight into the uptake pathway. Figure 3 shows cross-sectional TEM images of CHO-β cells cultured with SiNW-NH_2_ and fixed at 2, 3, 5, and 10 h. It represents snapshots of the internalization process at 37 °C and highlights how the membrane of the cells interacts with the wires. In the first 2 h, the wires were just starting to interact with the cell membrane via a non-specific charge–charge interaction process. In Fig. 3a, the wire is observed to be tangent with the membrane, but not yet entering, showing the binding stage of the internalization process. After 3 h of incubation, the membrane is shown changing its shape in Fig. 3b in order to accommodate the wires. After 5 and 10 h, depicted in Fig. 3c, d, respectively, the membrane has closely wrapped around the wires and pockets have formed, with an average gap of 18 nm between the wires and the membrane, as a means to “swallow” the wires. The formation of the pockets and the fact that the wires were not to target a specific receptor confirms that the uptake pathway of the SiNW-NH_2_ at 37 °C is via the membrane wrapping mechanism.

**Fig. 4:**
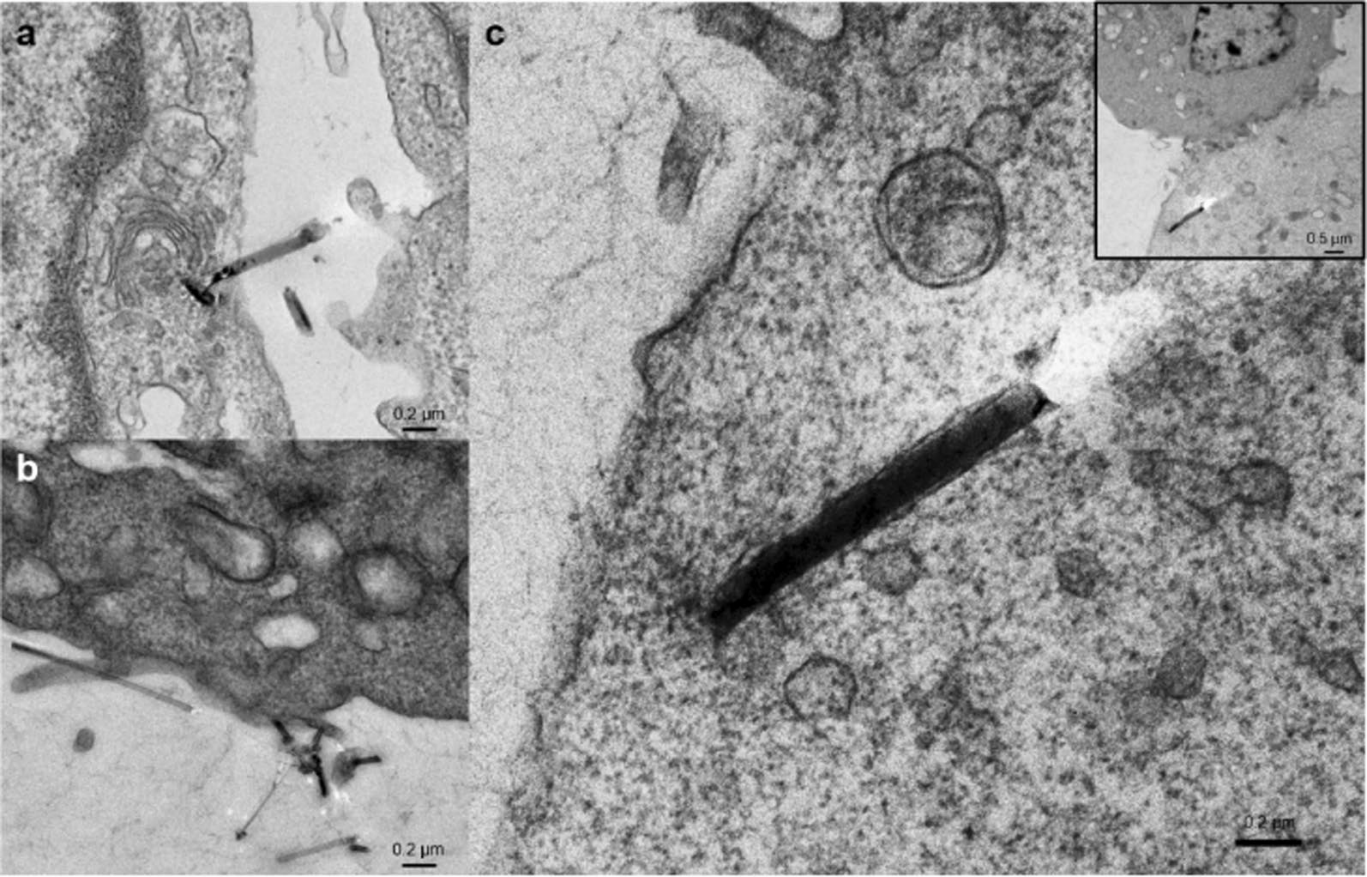
Cross section TEM micrographs represent CHO-β cells incubated with SiNW-NH_2_ for **a** 2, **b** 12, **c** 24 h at 4 °C. *Inset* in **c** represents the lower magnification image of the wire in **c**. *Scale bars* are 0.2 µm in **a**–**c** and 0.5 µm in the inset, respectively

To confirm the internalization of SiNW-NH_2_ is also occurring at a lower temperature than 37 °C, we performed the cross-sectional TEM experiment at 4 °C. The SiNW-NH_2_ were co-cultured with cells in 4 °C and fixed at different incubation periods up to 24 h. The uptake and internalization of the SiNW-NH_2_ at 4 °C is depicted in Fig. 4. The key steps during this process, including the initial interaction induced wire-membrane binding (Fig. 4a), the membrane accommodating the wires (Fig. 4b), and the internalization with the formed pocket of membrane closely surrounding the wires (Fig. 4c) are observed at 4 °C. Representative images obtained indicate that the binding is consistent with the optical microscopy data in Fig. 2. The internalization was confirmed at 24 h incubated samples at 4 °C, later than the observation from optical and fluorescence images, which could be attributed to limitation of the TEM cross section sample preparation and small sampling acquired through TEM. In summary, the temperature independent observations of the internalization and the formation of the membrane pockets are strong evidence to support the membrane wrapping mechanism and that this mechanism is, indeed, due to a physical force exerted on the membrane by the wires.

### Confocal fluorescence confirms the physical membrane wrapping

**Fig. 5:**
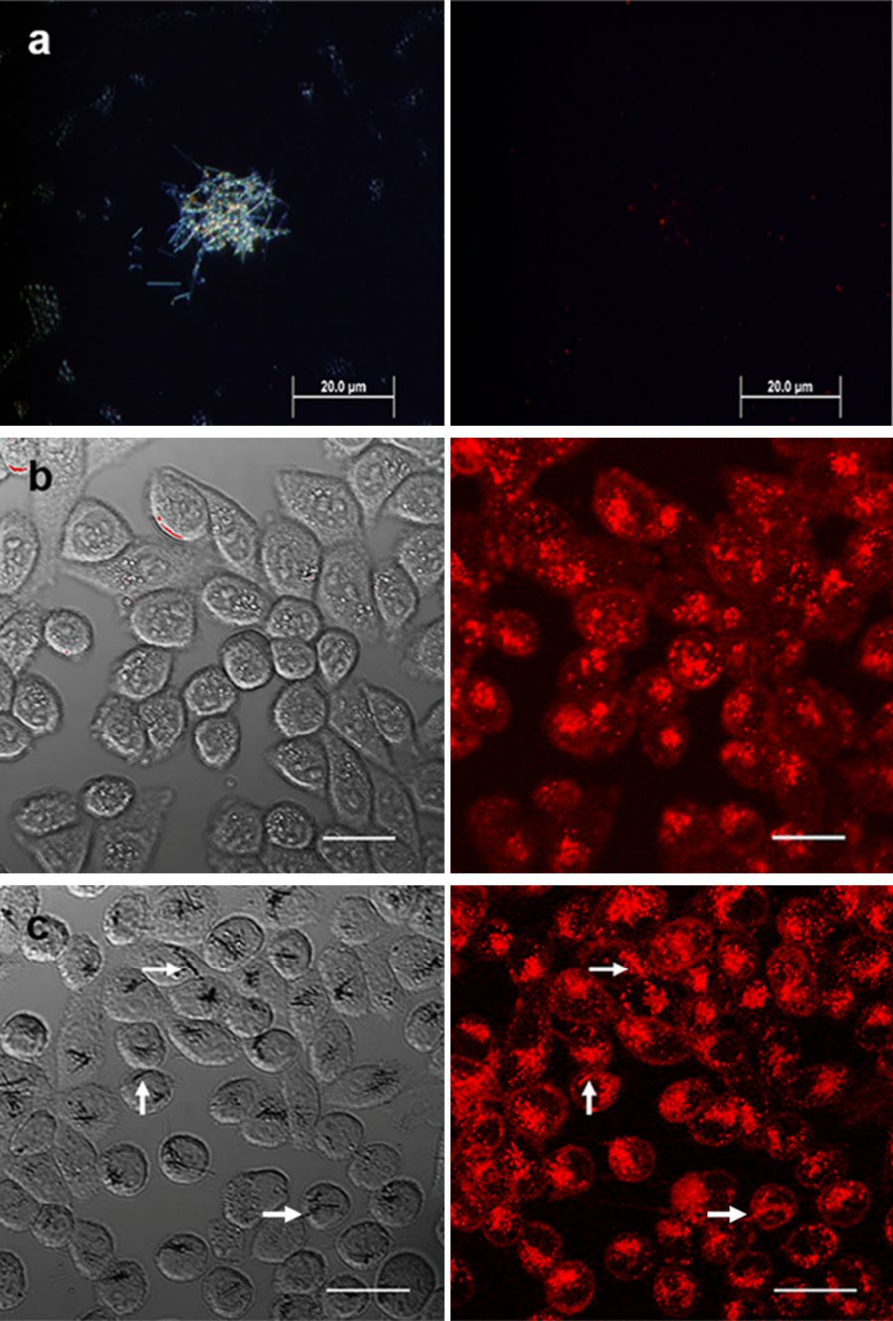
Confocal images of SiNWs after incubation with CHO-β cells. **a** Dark field (*left*) and fluorescence (*right*) images of the SiNW-NH_2_ after 1 h of incubation with DiI at 37 °C. **b** The transmission and fluorescence images of the control and **c** after 10 h of incubation with SiNW-NH_2_ at 37 °C. *Arrows* represent location of wires. *Scale bars* 20 µm

We performed confocal fluorescence microscopy on the cell samples cultured with SiNW-NH_2_, in which 3D information about nanowires and cells can be obtained through scanning along the z-axis and used to confirm the internalization of the wires. DiI (red) was used to stain the cell membrane. First, Fig. 5a shows a control study in which SiNWs were incubated with DiI in the cell medium without the presence of the cells. After one hour of incubation, the dark field image (Fig. 5a left) shows the presence of wires and the fluorescence image confirms that the wires do not fluoresce, suggesting the DiI does not label the surface of nanowires.

Figure 5b, c show bright field and fluorescence images obtained from the control group without wires (Fig. 5b) and the CHO-β cells co-cultured with SiNW-NH_2_ for 10 h at 37 °C (Fig. 5c). Bright field optical images on the left column of Fig. 5 show the presence of wires and the fluorescence images on the right column were used to locate the cell membrane, therefore indicating the interaction between wires and cellular membrane. Specifically, since the images plotted here were obtained towards the center of the top and bottom of the cells along the z scanning under the confocal microscope, the wires shown in focus in the bright field are in the focal plane therefore considered to be inside the cell. Clearly different from Fig. 5b, c demonstrated internalization of SiNW-NH_2_ by CHO-β cells. Importantly, Fig. 5c also shows that wire-shaped fluorescent signals present in the cells, indicating the internalized SiNW-NH_2_ became fluorescent. Together with observation that SiNWs were not directly labeled by Dil from Fig. 5a, these results also suggest that internalized SiNW-NH_2_ is through membrane wrapping. More fluorescence images obtained from 2- and 5-h incubation periods along with a plot showing average number of wires per cell internalized as a function of incubation time period are included in Additional file 1 (Figures S5, S6, respectively).

**Fig. 6:**
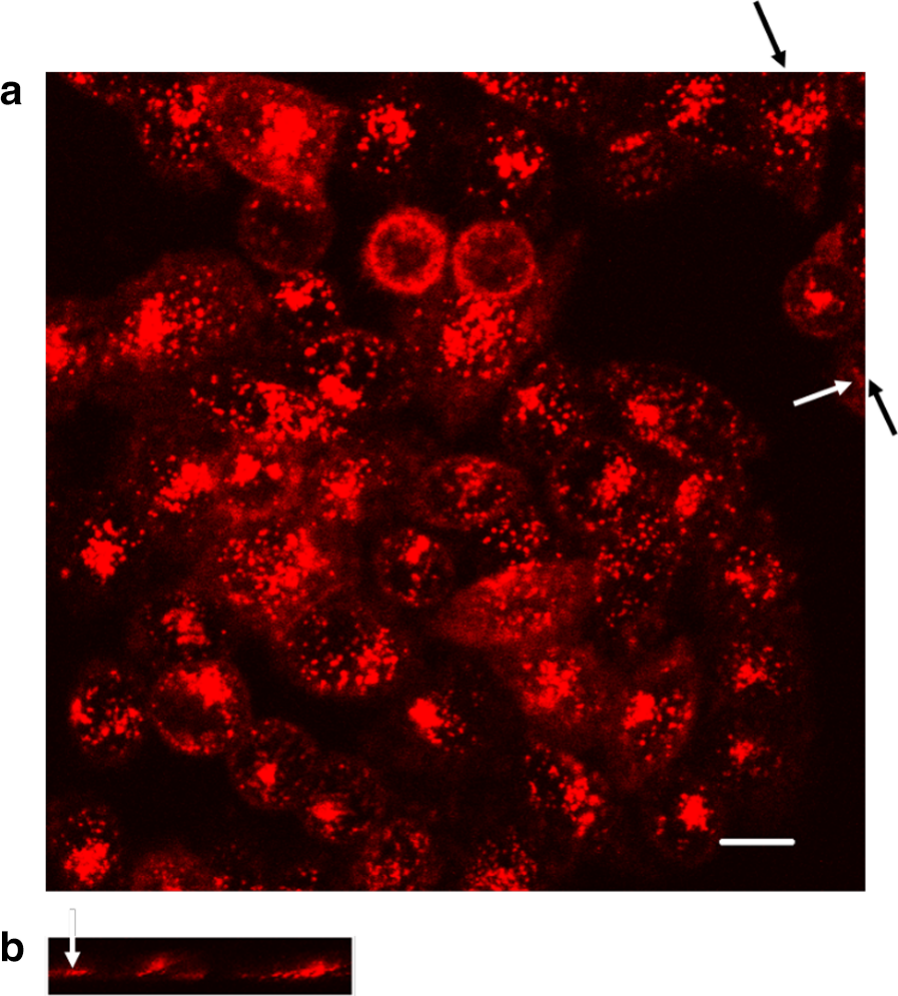
Confocal images of SiNW-NH_2_ after incubation with HeLa cells. Top (**a**) and orthogonal (**b**) views of the fluorescence images. The *white arrow* highlights the wire. The *black arrows* indicate the position where the orthogonal view was taken. *Scale bar* is 10 µm

The confocal experiment was also performed using the immortalized HeLa cell line using both unmodified SiNWs and SiNW-NH_2_. The results showed that, much like we saw with the CHO-β cells previously [22], the unmodified silicon nanowires did not interact with the cells, even after 5 h at both 37 and 4 °C (Additional file 1: Figures S7, S8). For amine-modifed SiNWs, Fig. 6 shows top and orthogonal views, demonstrating the co-localization of amine-modified SiNWs with cell membranes after 2 h of incubation at 37 °C, which is consistent with our findings with CHO-β cells. These results demonstrate that the SiNW-NH_2_ are internalized via a physically driven, membrane wrapping pathway, rather than an energy-dependent, receptor-mediated process.

## Conclusion

**Scheme 1:**
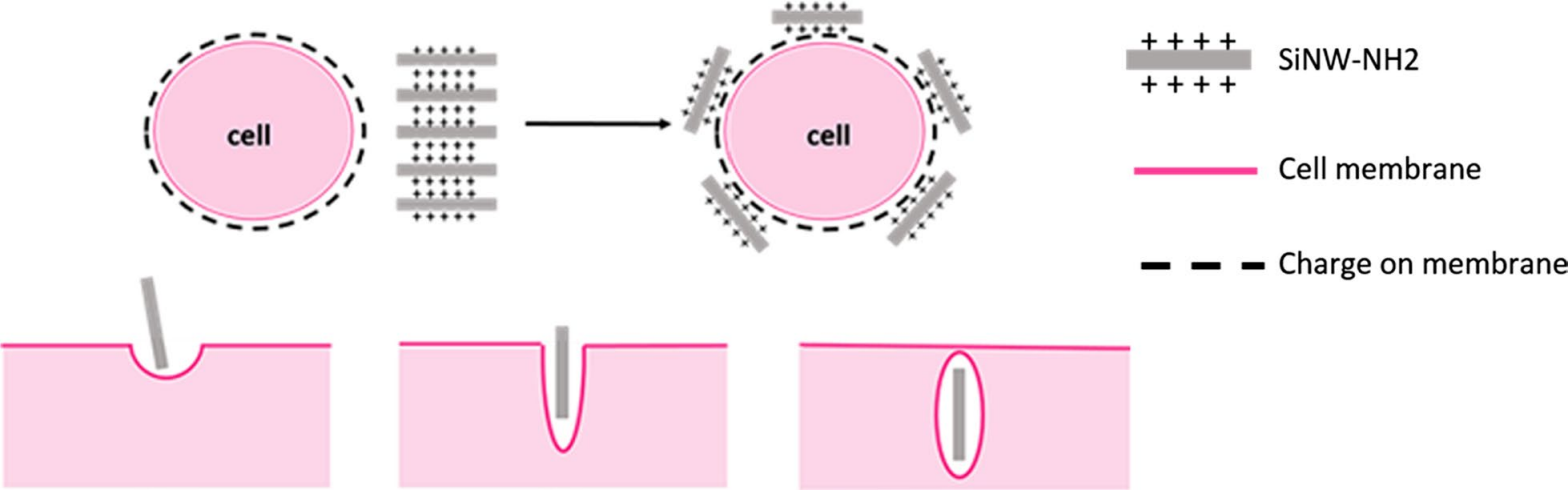
Schematic illustration demonstrating the membrane wrapping mechanism of the amine-functionalized SiNWs visualized

We have successfully visualized the key steps of internalization of SiNW-NH_2_ into cells as illustrated in Scheme 1, using optical, confocal fluorescence and transmission electron microscopies. Specifically, we confirmed that SiNW-NH_2_ are internalized by CHO-β and HeLa cells via a membrane wrapping mechanism for the first time and that this process is most likely due to physical force exerted on the membrane. Optical microscopy images at 37 °C incubation as well as 4 °C incubation show that the charge–charge induced binding and interactions occur regardless of temperature, indicating that the mechanism is physical rather than receptor mediated. Further, TEM micrographs captured the key steps of interactions between the cell membrane and the wires showing that the internalized wires are surrounded by pocket of the membrane. The confocal images independently demonstrate that the wires are successfully internalized. Fluorescence of the wires internalized confirms that the mechanism is membrane wrapping. This work provides better understanding of cellular uptake pathways for 1D nanomaterials without specific targeting ligands. Since the interaction presented here is physical, this work opens up the potential for using 1D materials to understand membrane mechanics through physical stress exerted on the membrane.

## Additional file

**Additional file 1.** Wire characterization including representative TEM of unmodified SiNWs and SiNW-NH_2_, FTIR analysis of unmodified SiNWs and SiNW-NH_2_, and wide XPS spectrum for an amine-modified silicon nanowires. Confocal images of SiNW-NH_2_ after incubation with CHO-β cells at 2 and 5-hour incubation periods and a graph plotting the average number of wires internalized per cell as a function of incubation time at 37 °C. Confocal images of HeLa cells at 2 and 5-h incubation time at 37 and 4 °C with unmodified SiNWs.

## Abbreviations

1D: one-dimensional
SiNWs: silicon nanowires
SiNW-NH_2_: amine-functionalized silicon nanowires
TEM: transmission electron microscopy
0D: zero dimensional
SWCNTs: single-walled carbon nanotubes
CVD: chemical vapor deposition
CHO-β: Chinese hamster ovary cells transfected with folate receptor beta
APTMS: (3-aminopropyl) trimethoxysilane
RPMI: Roswell Park Memorial Institute
PBS: phosphate buffered saline
DiI: 1,1′-dioctadecyl-3,3,3′,3′-tetramethylindocarbocyanine perchlorate
FTIR: Fourier transform infrared spectroscopy
XPS: X-ray photoelectron spectroscopy
