# Supplementary material for: Understanding cellular internalization pathways of silicon nanowires
Source: J Nanobiotechnology. 2017 Mar 1;15:17. doi: 10.1186/s12951-017-0250-0 (PMC5333412; doi:10.1186/s12951-017-0250-0)
Supplement: Supplementary file 1 — Additional file 1. Wire characterization including representative TEM of unmodified SiNWs and SiNW-NH2, FTIR analysis of unmodified SiNWs and SiNW-NH2, and wide XPS spectrum for an amine-modified silicon nanowires. Confocal images of SiNW-NH2 after incubation with CHO-β cells at 2 and 5-hour incubation periods and a graph plotting the average number of wires internalized per cell as a function of incubation time at 37 °C. Confocal images of HeLa cells at 2 and 5-h incubation time at 37 and 4 °C with unmodified SiNWs. [file 12951_2017_250_MOESM1_ESM.docx]

**Supporting Information**

**Investigation of Cellular Internalization Pathways of Surface-modified Silicon Nanowires**

Kelly McNear^1^, Yimin Huang^1^, Chen Yang^*,1,2^

^1^Department of Chemistry, Purdue University, West Lafayette, Indiana 47907, United States

^2^Department of Physics and Astronomy, Purdue University, West Lafayette, Indiana 47907, United States

^*^Corresponding Author: yang@purdue.edu

**
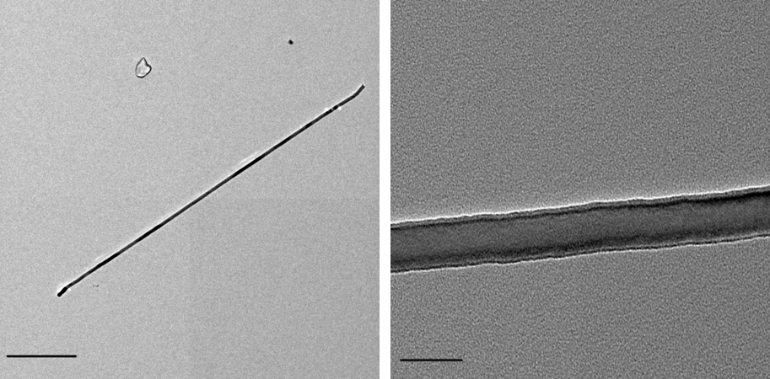
**

**Figure S1.** Representative TEM micrograph of pure SiNW-NH_2_. The scale bars are 1 µm (left) and 50 nm (right) The dimension was found to be 40.1 nm in diameter and 5.5 µm in length, which is consistent with the expected values from the growth condition.

**
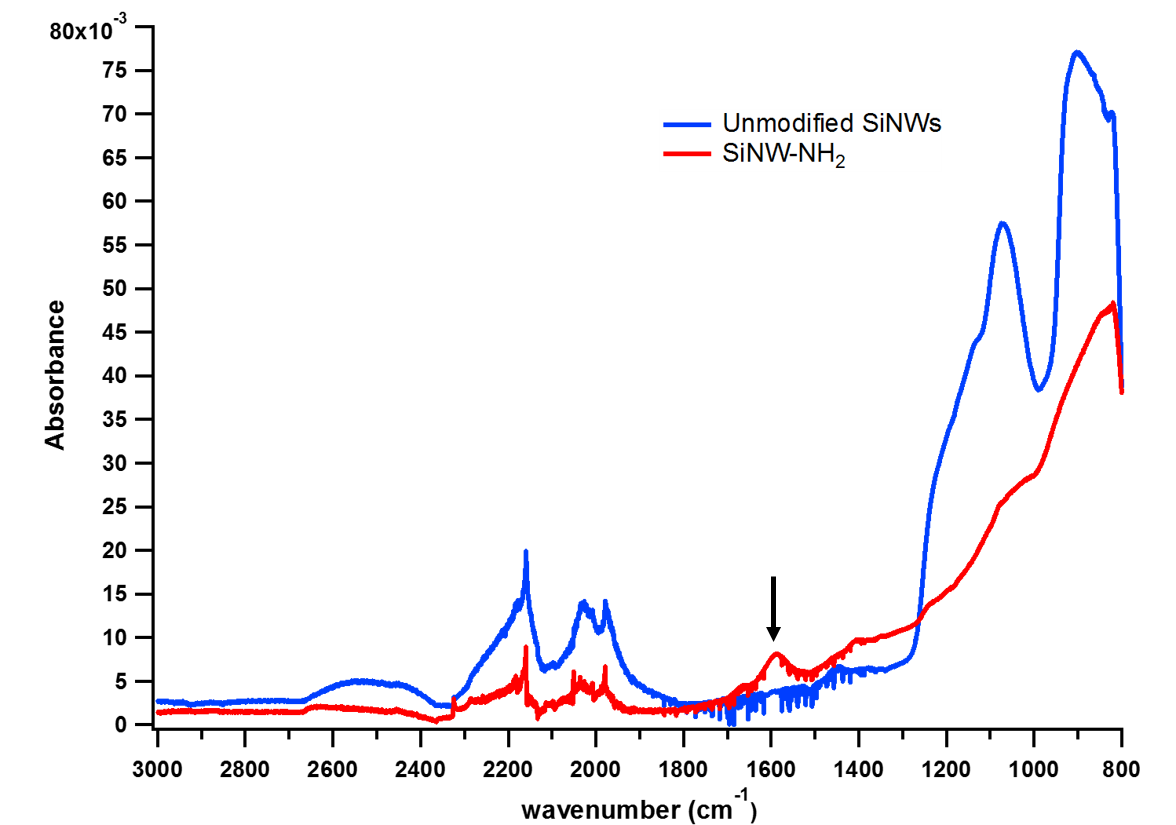
**

**Figure S2.** Representative FTIR analysis of the unmodified SiNWs (blue) and the SiNW-NH_2_ (red). Arrow indicates NH_2_ bending peak present in for the modified wires.

**
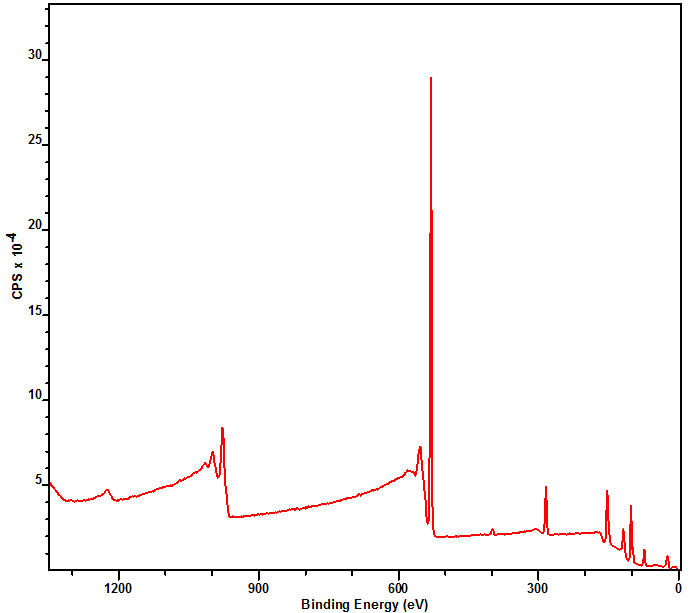
**

**Figure S3.** Representative XPS analysis of an amine-modifed silicon nanowires.

**
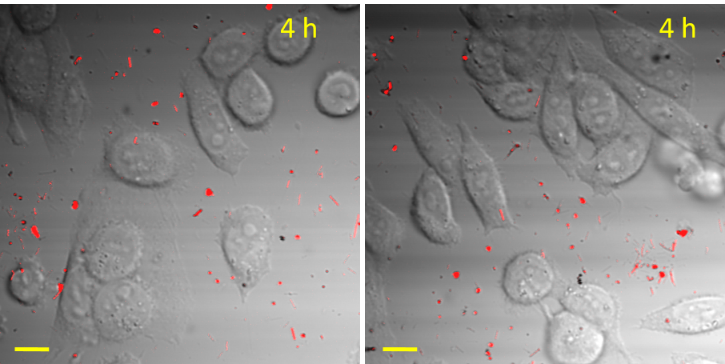
**

**Figure S4.** Representative images of SiNWs (red) with CHO-β cells. Overlay of four-wave mixing and transmission images of unmodified SiNWs after incubation for 4 h. Scale bars, 10 µm.

**
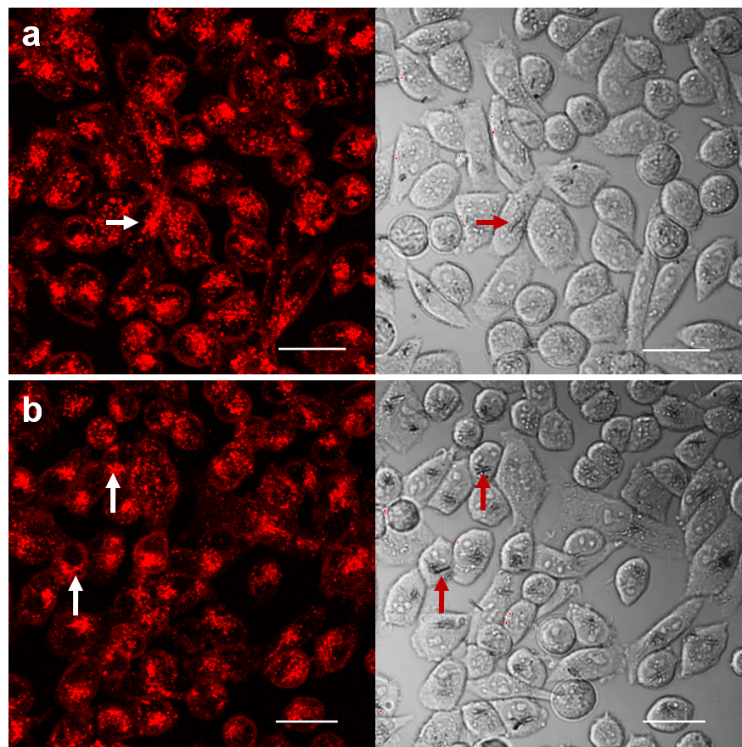
**

**Figure S5. Confocal images of SiNW-NH_2_** at 37 °C incubation with CHO-β cells. (a,b) The fluorescence and transmission images of cells after 2 and 5 hours incubation of SiNW-NH_2_ at 37 °C, respectively. Arrows represent location of wires. Scale bars, 20 µm.


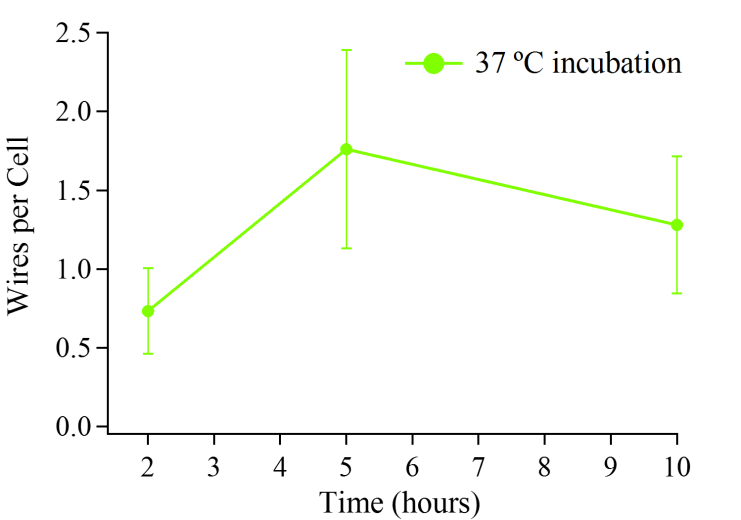


**Figure S6.** The number of wires internalized per cell as a function of incubation time at 37 °C (green). Five areas, each measuring 32 × 28 µm, from each image were counted and the standard deviation is presented with error bars.


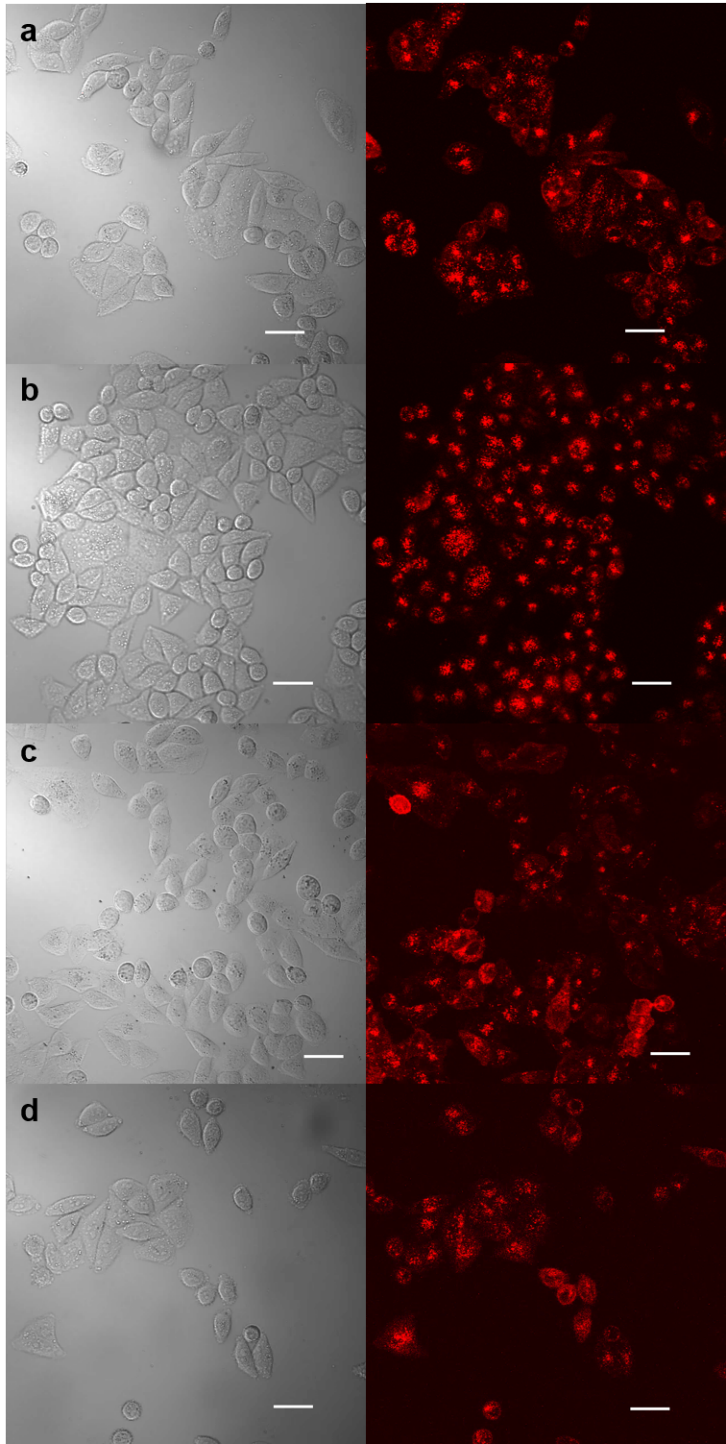


**Figure S7.** Confocal images of unmodified SiNWs after incubation at 37 °C with HeLa cells. **a**) The transmission and fluorescence images of the control, **b**) after 2 hours of incubation with unmodified SiNW, **c**) after 3 hours of incubation with SiNW and **d**) after 5 hours of incubation with SiNW. Scale bars, 100 µm.

**
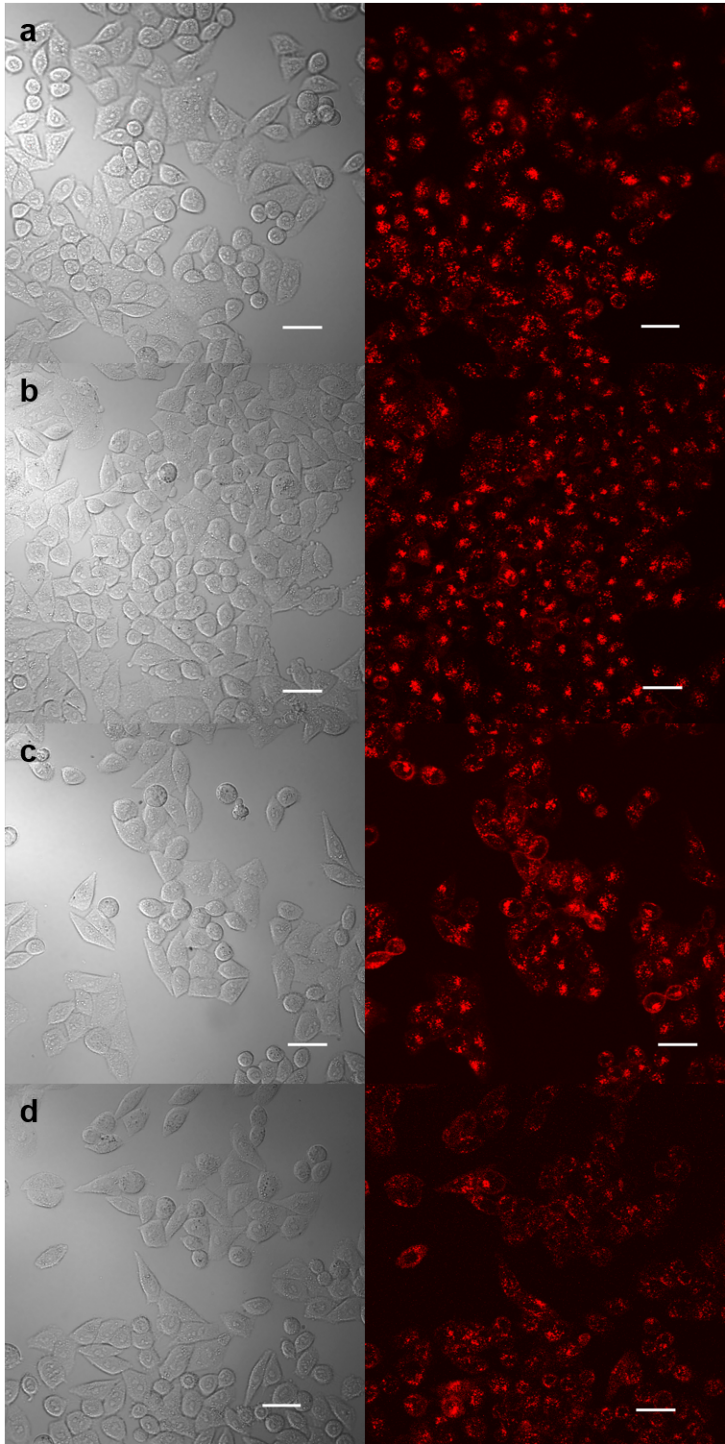
**

**Figure S8.** Confocal images of unmodified SiNWs after incubation at 4 °C with HeLa cells. **a**) The transmission and fluorescence images of the control, **b**) after 2 hours of incubation with unmodified SiNW, **c**) after 3 hours of incubation with SiNW and **d**) after 5 hours of incubation with SiNW. Scale bars, 100 µm.
